# Prognostic factors for gastric cancer patients aged ≥ 85 years

**DOI:** 10.1186/s12885-024-12512-2

**Published:** 2024-06-18

**Authors:** Shunji Endo, Masaharu Higashida, Kei Furuya, Shuya Yano, Toshimasa Okada, Kazuhiko Yoshimatsu, Yoshinori Fujiwara, Tomio Ueno

**Affiliations:** Department of Digestive Surgery, Kawasaki Medical School, 577, Matsushima, Kurashiki, 701-0192 Okayama Japan

**Keywords:** Aged, 85 and over, Gastrectomy, Prognosis, Stomach neoplasms

## Abstract

**Background:**

As gastric cancer patients aged ≥ 85 years have a short life expectancy and often die from other diseases such as pneumonia, indications for surgery are controversial. In this study, we retrospectively analyzed the prognostic factors of elderly patients with gastric cancer who are candidates for curative gastrectomy.

**Methods:**

Among 114 patients aged ≥ 85 years with gastric cancer at our hospital between 2010 and 2019, prognostic factors were examined using the Cox proportional hazards model in 76 patients excluding those with cStage IVB or endoscopic submucosal dissection. We also analyzed the factors of pneumonia death.

**Results:**

cStage was I/IIA/IIB/III/IVA in 37/6/14/14/5 patients, respectively. Treatment included distal gastrectomy in 28 patients, total gastrectomy in 6, local resection in 9, others in 3, and no surgery in 30. In univariate analyses of overall survival, Eastern Cooperative Oncology Group Performance Status, physiological score of Physiological and Operative Severity Score for the enUmeration of Mortality and morbidity (POSSUM), Onodera’s prognostic nutritional index, cStage, and treatment were prognostic factors. In a multivariate analysis, POSSUM physiological score, cStage, treatment method {no surgery vs. distal gastrectomy: hazard ratio (HR) 5.78, 95% confidence interval (CI) 2.33–14.3}, (total gastrectomy vs. distal gastrectomy: HR 4.26, 95% CI 1.22–14.9) were independent prognostic factors. In univariate analyses of pneumonia-specific survival, treatment (total gastrectomy vs. distal gastrectomy: HR 6.98, 95% CI 1.18–41.3) was the only prognostic factor.

**Conclusions:**

The prognosis of distal gastrectomy was better than that of non-surgery even in patients aged ≥ 85 years. However, total gastrectomy was considered to be avoidable due to the high rate of postoperative pneumonia death.

## Background

Gastric cancer is a major cancer, ranking fifth in the number of incidences (1,089,103) and fourth in the number of deaths (768,793) among all cancers worldwide in 2020 [1]. In Japan, the numbers are on the decline; however, it ranked third in the number of incidences (124,319) in 2019 and third in the number of deaths (41,624) in 2021 [2]. Furthermore, as the population ages, the proportion of elderly patients with gastric cancer is increasing.

According to statistics in 2022, the average life expectancy of Japanese people was 81.05 years for men and 87.09 years for women [3]. Thus, doctors are encountering more gastric cancer patients aged ≥ 85 years. In 2019, the number of incidences of gastric cancer patients aged ≥ 85 years in Japan was 20,600, accounting for 16.6% of the total, and in 2021, the number of gastric cancer deaths was 13,773, accounting for 33.1% of the total [2].

People aged ≥ 85 years are sometimes referred to as the oldest-old [4]. Among this demographic, those diagnosed with gastric cancer commonly exhibit frailty, malnourishment, declining physical and cognitive functions, comorbidities, and a short life expectancy. The average life expectancy of a Japanese person aged 85 is 6.20 years for men and 8.28 years for women [3]. Thus, the treatment approach for patients aged ≥ 85 years may need to be distinct from that used for younger patients.

The first-choice treatment for gastric cancer that is not amenable to endoscopic submucosal dissection (ESD) is radical gastrectomy, but performing a gastrectomy in exchange for curing the cancer reduces quality of life (QOL) and carries the risk of complications, which may ultimately lead to a shortened life period, and is therefore not necessarily optimal for the very elderly. In clinical practice, patients, their families, and doctors often wonder whether they should undergo surgery. If they undergo surgery, determining the most beneficial surgical procedure for this demographic is important.

In this study, we retrospectively analyzed prognostic factors of patients aged ≥ 85 years with curable gastric cancer to verify whether it is appropriate to inflict the invasive step of gastrectomy in exchange for a radical cure.

## Methods

### Patients

**Fig. 1:**
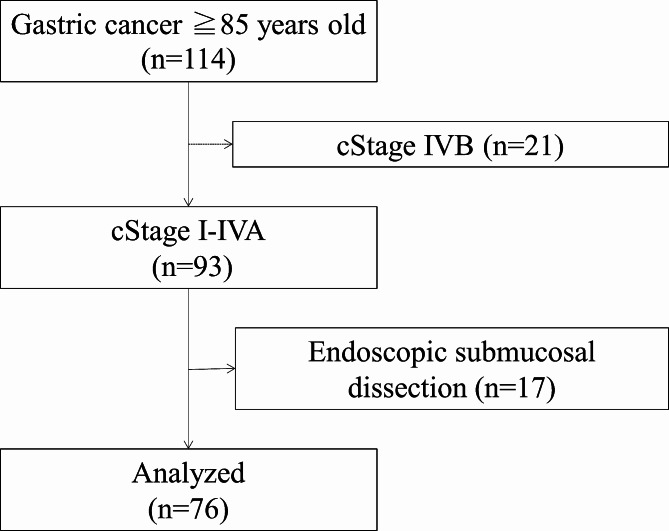
The participant flow diagram

At our institution, 114 patients aged ≥ 85 years were diagnosed with gastric cancer on the basis of histopathological examinations between 2010 and 2019. We excluded 21 patients with clinical stage IVB cancer according to the Japanese Classification of Gastric Carcinoma (15th edition) [5], and 17 patients who underwent ESD since they were not subject to the discussion of whether to undergo gastrectomy. We retrospectively reviewed 76 patients. A flowchart of patient selection is shown in Fig. 1.

Information about the following characteristics and clinical parameters at diagnosis were collected from the patients’ medical records: age, sex, body mass index, Eastern Cooperative Oncology Group Performance Status (ECOG PS) score [6], Physiological and Operative Severity Score for the enUmeration of Mortality and morbidity (POSSUM) physiological score [7], Onodera’s prognostic nutritional index (PNI) [8], clinical stage of gastric cancer, and treatment. POSSUM physiological score was calculated based on the patient’s age, cardiac signs, chest radiography signs, respiratory history, systolic blood pressure, pulse rate, Glasgow coma scale score, hemoglobin level, white blood cell count, plasma urea level, plasma sodium level, plasma potassium level, and electrocardiography results. Each item was scored from 1 (normal) to 8 (abnormal). Adding all the scores together gave a physiological score ranging from a minimum of 12 to a maximum of 88, with a higher score indicating higher surgical risk. PNI was calculated using the following formula: 10 × serum albumin (g/dL) + 0.005 × total lymphocyte count (/mm^3^). The clinical stages of gastric cancer were evaluated by the attending surgeon using esophagogastroduodenoscopy and chest and abdominal contrast CT scans (or plain CT), and the final decision was made at conferences of surgeons and gastroenterologists according to the Japanese Classification of Gastric Carcinoma (15th edition). Surgical procedures adhered to the Japanese Gastric Cancer Treatment Guidelines 2021 (6th edition) [9]. Prognoses, including the last date known to be alive or the date of death and its cause, were gathered from medical records housed at our institution or referral institutions, or by calling the patients or their families.

### Statistical analysis

Continuous variables were compared using the Mann–Whitney *U* test. Categorical variables were compared using the chi-square test or Fisher’s exact probability test. Overall survival (OS) was defined as the interval from the date of cancer diagnosis to the date of death from any cause. Surviving patients were censored at the date that they were last known to be alive. Pneumonia-specific survival was defined as the interval from the date of cancer diagnosis to the date of death from pneumonia. Surviving patients and those who died from causes other than pneumonia were censored. Hazard ratios for death were estimated using Cox regression analysis. Survival was shown on Kaplan–Meier curves. Analyses were performed using JMP software (version 14.2.0 for Windows; SAS Institute Inc., Cary, NC, USA).

### Disclosure of ethical statement

The protocol for this research project was approved by the Institutional Review Board of Kawasaki Medical School (approval number 5083-01) and conformed to the Declaration of Helsinki’s provisions.

## Results

Patients’ characteristics are summarized in Table 1. Treatment included distal gastrectomy for 28 patients, total gastrectomy for six patients, proximal gastrectomy for one patient, local resection for nine patients, gastrojejunostomy for one patient, and probe laparotomy for one patient. Eight of these patients underwent laparoscopic surgery. Thirty patients were deemed to be curably resectable; however, surgery was not performed. The rationale for no surgery included nine patient refusals, 13 family refusals, and eight doctor decisions. Patients who did not undergo surgery had significantly worse PS and PNI than those who underwent surgery. There was no significant difference in cancer stage. Except for one patient who underwent probe laparotomy due to peritoneal dissemination and was administered oral S-1, none of the other patients received chemotherapy.

**Table 1:** Patients’ characteristics

Variables		Total	Surgery	No surgery	*p*
		(*n* = 76)	(*n* = 46)	(*n* = 30)	
Age, years	Median (Range)	87 (85–96)	86.5 (85–92)	88 (85–96)	0.31
Sex, n (%)	Male	48 (63)	18 (64)	17 (57)	0.34
ECOG PS, n (%)	0	14 (18)	13 (28)	1 (3)	< 0.01
	1	26 (34)	17 (37)	9 (30)	
	2	13 (17)	9 (20)	4 (13)	
	3	17 (22)	6 (13)	11 (37)	
	4	5 (7)	1 (2)	4 (13)	
	unknown	1 (1)	0	1 (3)	
POSSUM physiological score	Median (Range)	30.5 (20–46)	29 (20–44)	34 (21–46)	0.10
PNI	Median (Range)	41.3 (19.0-56.4)	42.8 (19.1–56.4)	38.8 (19.0–52.0)	0.02
cStage, n (%)	I	37 (51)	21 (46)	16 (53)	0.52
	IIA/IIB	6(8)/14(18)	3(7)/10(22)	3(10)/4(13)	
	III	14 (18)	9 (20)	5 (17)	
	IVA	5 (6)	3 (7)	2 (7)	
pStage, n (%)	IA/IB		16(35)/1(2)		-
	IIA/IIB		10(22)/5(11)		
	IIIA/IIIB/IIIC		4(9)/3(7)/1(2)		
	IV		6 (13)		

*ECOG PS* Eastern Cooperative Oncology Group Performance Status, *POSSUM* Physiological and Operative Severity Score for the enUmeration of Mortality and morbidity, *PNI* Onodera’s prognostic nutritional index

At the time of analysis, 63 patients had died. The median follow-up period of the surviving patients was 66.0 months. The median OS time was 31.6 months, and the five-year OS rate was 28.0%. The causes of death are shown in Table 2. Although there was no significant difference in known causes of death between the surgery and no surgery groups, the leading cause in the no surgery group was gastric cancer whereas the leading cause in the surgery group was pneumonia.

**Table 2:** Causes of death

Causes	Total	Surgery	No surgery	*p*
	(*n* = 76)	(*n* = 46)	(*n* = 30)	
Gastric cancer	21	10	11	0.15
Pneumonia	15	11	4	0.26
Other malignancies	4	3	1	1.00
Stroke	4	4	0	0.15
Sudden death	3	3	0	0.27
Others	5	3	2	1.00
Unknown	11	1	10	< 0.01
Alive	13	11	2	0.05

Other malignancies included hepatocellular carcinoma, cholangiocarcinoma, rectal cancer (surgery group), and colon cancer (no surgery group). Others included pleurisy, chronic obstructive pulmonary disease, heat stroke (surgery group), heart failure, and senility (no surgery group).

In univariate analyses for OS, ECOG-PS (3 vs. 0), POSSUM physiological score (≥ 30 vs. ≥ 20, ≤ 29), PNI (< 45 vs. ≥ 45), clinical stage (IIA vs. I), treatment method (total gastrectomy, no surgery vs. distal gastrectomy) were significantly correlated with worse mortality outcomes (Table 3). Multivariate analysis was conducted using these significant factors, and revealed that POSSUM physiological score (≥ 30 vs. ≥ 20, ≤ 29), clinical stage (III vs. I), and treatment method (total gastrectomy, no surgery vs. distal gastrectomy) were independent risk factors for mortality.

**Table 3:** Univariate and multivariate analyses for overall survival

Valuables		Univariate analysis				Multivariate analysis		
		HR	(95%CI)	*p*		HR	(95%CI)	*p*
Age (yr)	≥ 85, < 89	Reference						
	≥ 90	1.37	(0.76–2.47)	0.29				
Sex	male	Reference						
	female	1.27	(0.75–2.16)	0.37				
BMI	≥ 18.5, < 25	Reference						
	< 18.5	0.89	(0.43–1.84)	0.76				
	≥ 25	0.40	(0.14–1.13)	0.08				
ECOG PS	0	Reference				Reference		
	1	1.84	(0.89–3.80)	0.11		1.99	(0.84–4.59)	0.12
	2	0.85	(0.34–2.13)	0.73		0.68	(0.23–2.04)	0.49
	3	2.67	(1.20–5.91)	0.02		1.24	(0.43–3.58)	0.69
	4	2.46	(0.84–7.20)	0.10		0.79	(0.19–3.25)	0.74
POSSUM physiological score	≥ 20, ≤ 29	Reference				Reference		
	≥ 30, ≤ 39	1.93	(1.10–3.38)	0.02		2.47	(1.15–5.29)	0.02
	≥ 40	3.16	(1.43–7.01)	< 0.01		4.79	(1.83–12.5)	< 0.01
PNI	≥ 45	Reference				Reference		
	≥ 40, < 45	2.46	(1.21-5.00)	0.01		1.78	(0.74–4.31)	0.20
	< 40	2.73	(1.39–5.34)	< 0.01		1.41	(0.55–3.61)	0.47
cStage	I	Reference				Reference		
	IIA	2.99	(1.21–7.40	0.02		2.01	(0.59–6.87)	0.26
	IIB	1.29	(0.64–2.60)	0.48		1.84	(0.70–4.79)	0.21
	III	1.69	(0.87–3.25)	0.12		3.26	(1.30–8.18)	0.01
	IVA	2.18	(0.76–6.24)	0.15		2.70	(0.81–9.06)	0.11
Treatment	Distal gastrectomy	Reference				Reference		
	Total gastrectomy	4.72	(1.81–12.4)	< 0.01		4.26	(1.22–14.9)	0.02
	Local resection	0.62	(0.23–1.65)	0.34		1.78	(0.50–6.34)	0.38
	No surgery	3.37	(1.80–6.29)	< 0.01		5.78	(2.33–14.3)	< 0.01

*BMI* body mass index, *ECOG PS* Eastern Cooperative Oncology Group Performance Status, *POSSUM* Physiological and Operative Severity Score for the enUmeration of Mortality and morbidity, *PNI* Onodera’s prognostic nutritional index, *HR* hazard ratio, *CI* confidence interval

**Fig. 2:**
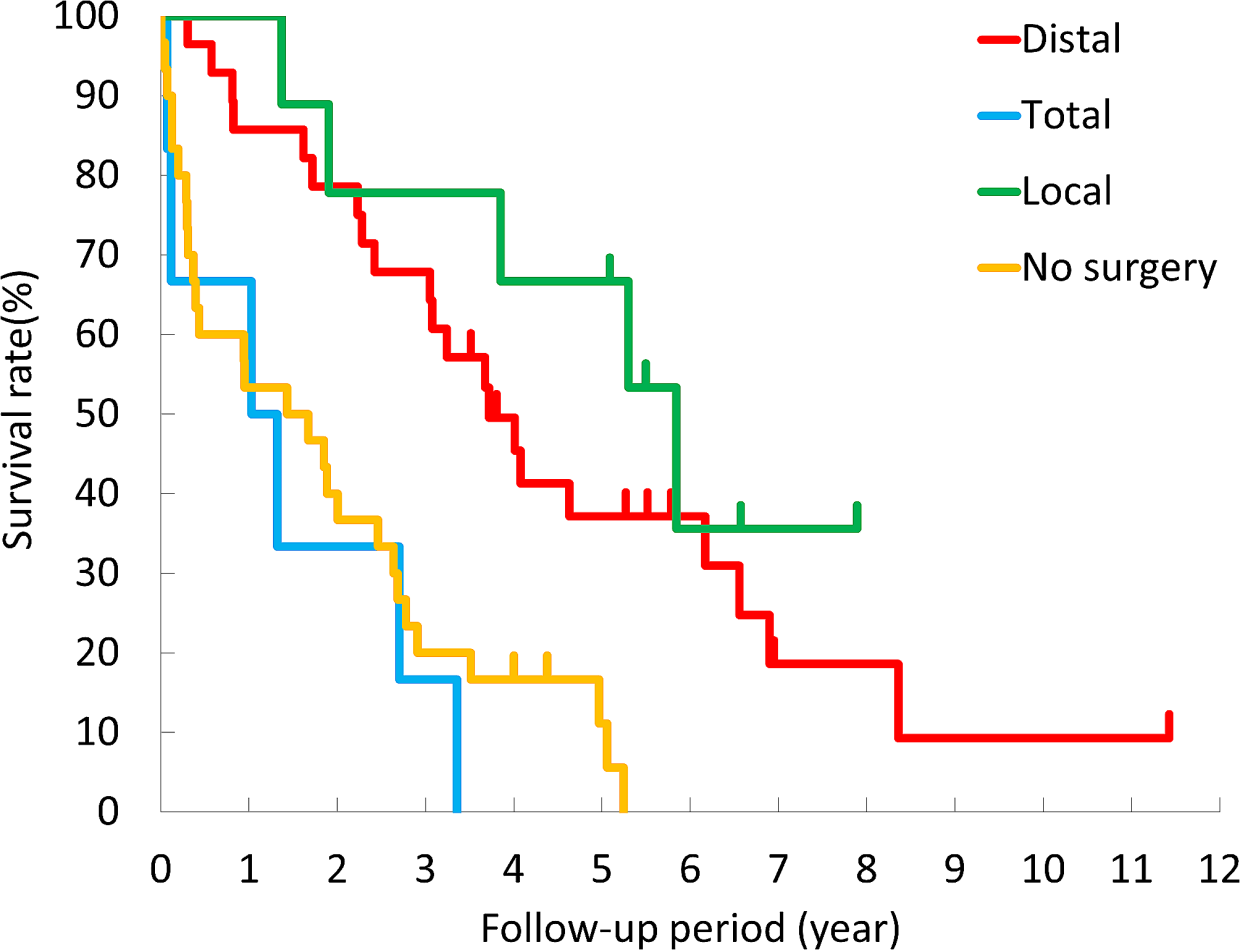
Kaplan–Meier overall survival curves of gastric cancer patients aged ≥ 85 years by treatment method (extent of gastrectomy)

Overall survival curves by treatment method are shown in Fig. 2. The prognosis was most favorable following local resection; however, there was no significant difference compared to the prognosis after distal gastrectomy (*p* = 0.33). The prognosis after distal gastrectomy was significantly more favorable than after total gastrectomy (*p* < 0.01); however, there was no significant difference between the prognosis after total gastrectomy and without surgery (*p* = 0.49).

In univariate analyses for pneumonia-specific survival, treatment method (total gastrectomy vs. distal gastrectomy) was the only prognostic factor (Table 4).

**Table 4:** Univariate analyses for pneumonia-specific survival

Valuables		Univariate analysis		
		HR	(95%CI)	*p*
Age (yr)	≥ 85, < 89	Reference		
	≥ 90	2.23	(0.74–6.69)	0.15
Sex	male	Reference		
	female	0.39	(0.09–1.78)	0.23
BMI	≥ 18.5, < 25	Reference		
	< 18.5	1.26	(0.33–4.75)	0.73
	≥ 25	0.48	(0.06–3.82)	0.49
ECOG PS	0	Reference		
	1,2	0.96	(0.28–3.31)	0.95
	3,4	1.69	(0.40–7.17)	0.48
POSSUM physiological score	≥ 20, ≤ 29	Reference		
	≥ 30, ≤ 39	2.78	(0.92–8.41)	0.07
	≥ 40	1.51	(0.17–13.2)	0.71
PNI	≥ 45	Reference		
	≥ 40, < 45	1.44	(0.38–5.43)	0.59
	< 40	1.29	(0.36–4.60)	0.69
cStage	I	Reference		
	IIA	2.40	(0.27-21.0)	0.43
	IIB	1.20	(0.31–4.66)	0.79
	III	1.35	(0.35–5.13)	0.66
	IVA	-	-	-
Treatment	Distal gastrectomy	Reference		
	Total gastrectomy	6.98	(1.18–41.3)	0.03
	Local resection	0.95	(0.18–4.93)	0.95
	No surgery	1.75	(0.45–6.80)	0.42

*BMI* body mass index, *ECOG PS* Eastern Cooperative Oncology Group Performance Status, *POSSUM* Physiological and Operative Severity Score for the enUmeration of Mortality and morbidity, *PNI* Onodera’s prognostic nutritional index, *HR* hazard ratio, *CI* confidence interval

## Discussion

In the present study, preoperative comorbidities (POSSUM physiological score), clinical stage, and surgical method were prognostic factors for oldest-old gastric cancer patients aged ≥ 85 years. The prognosis after distal gastrectomy was acceptable, whereas the prognosis after total gastrectomy was as poor as without surgery. This may be because patients often develop pneumonia after total gastrectomy.

POSSUM is a surgical risk scoring system proposed by Copeland et al. in 1991 [7], which calculates the incidence of postoperative complications and mortality by summing a 12-item physiological score and a 6-item operative severity score. Although it over-predicts postoperative mortality, various improved versions have since been developed and are still used today for risk assessment in gastrointestinal surgery. In the present study, the 12-item physiological score of the POSSUM scoring system was an independent prognostic factor for oldest-old gastric cancer patients. Care should be taken when deciding on treatment for patients with a score of 30 or higher.

We found that the survival curve after total gastrectomy almost overlapped with that without surgery. Thus, oldest-old people that underwent total gastrectomy did not receive survival benefit compared with those without surgery. Total gastrectomy may be more likely to cause aspiration pneumonia than distal gastrectomy, as patients easily experience jejunal content regurgitation into the esophagus after total gastrectomy because of the resection of the lower esophageal sphincter. Furthermore, since total gastrectomy causes greater weight loss than distal gastrectomy, emaciation and resulting sarcopenia may have an effect on prognosis. Recently, “sarcopenic dysphagia” is considered to be a cause of aspiration pneumonia [10]. Subtotal distal gastrectomy or near-total gastrectomy, which leaves a very small proximal stomach instead of total gastrectomy, has been reported for upper gastric cancer. Furukawa et al. [11] reported that patients who underwent laparoscopic subtotal gastrectomy had better nutritional status and no bile reflux than those who underwent laparoscopic total gastrectomy. Ko et al. [12] reported that patients who underwent laparoscopic near-total gastrectomy had improved nutritional status and QOL than those who underwent laparoscopic total gastrectomy. This procedure may be a better option for elderly patients with upper gastric cancer than total gastrectomy.

One of the causes of postoperative pneumonia is wound pain during breathing, and it has been suggested that the size and location of the wound may be related to pneumonia. Park et al. [13, 14] reported that totally laparoscopic gastrectomy had fewer pulmonary complications and better QOL than laparoscopy-assisted gastrectomy. Therefore, totally laparoscopic gastrectomy is preferable for the very elderly, who are at high risk of postoperative pneumonia. Unfortunately, at our institution, we did not perform totally laparoscopic gastrectomy during the study period, and only performed laparoscopy-assisted distal gastrectomy in five patients and laparoscopic local resection in three patients, with all remaining patients being performed by open surgery. Currently, we perform totally laparoscopic gastrectomy (including robotic surgery) in most patients, and we expect a decrease in postoperative pneumonia.

In this study, the prognosis was most favorable following local resection. However, it should be noted that all patients who underwent local resection had cStage I cancer, whereas 11 (39%) of those who underwent distal gastrectomy had cStage I cancer. For local resection, the hazard ratio for death was 0.62 in univariate analysis compared with distal gastrectomy, but the hazard ratio was 1.78 in multivariate analysis. Therefore, there is no evidence that local resection is sufficient instead of radical gastrectomy for elderly patients.

In recent years, there have been many articles discussing the safety and effectiveness of gastric cancer surgery for the elderly [15]; however, few studies focused on oldest-old patients aged ≥ 85 years. Takama et al. [16] reported that there were no significant differences in the frequency or grade of total complications or mortality between patients aged ≥ 85 years and those aged 75–84 years. Hikage et al. [17] reported that the overall postoperative complication rate was not significantly different between patients aged ≥ 85 years and those aged 75–84 years. Kiyokawa et al. [18] reported that gastrectomy with standard lymphadenectomy may be acceptable for relatively healthy patients aged ≥ 85 years.

The present study examined prognostic factors in both surgical and non-surgical patients aged ≥ 85 years. Our findings may be important as few papers have compared surgical and non-surgical cases in such patients. Endo et al. [19] reported that the prognosis was better after distal gastrectomy in patients aged ≥ 85 years than with best supportive care even after propensity score matching, but the prognosis after total gastrectomy was poor. Choo et al. [20] reported that patients aged ≥ 86 years with advanced gastric cancer did not show significantly better prognosis with surgical resection than with supportive care; therefore, they did not recommend surgery for such patients.

Because the purpose of this study was to resolve the dilemma of choosing between curing cancer and gastrectomy in oldest-old patients, we excluded ESD patients in which cancer treatment and stomach preservation could be achieved at the same time. ESD is considered to be a beneficial treatment for elderly patients, and a phase III trial to expand the indications of ESD to elderly patients is currently underway (JCOG1902) [21].

The present study had several limitations. First, it was limited by its retrospective nature. Second, it was conducted with a relatively small number of patients from a single institution. Especially due to the small number of patients who underwent laparoscopic gastrectomy, it was unable to analyze the impact of laparoscopy-assisted and totally laparoscopic procedures or open procedures. Third, some patients were not followed up for a sufficient period. In particular, most non-surgical patients did not visit our outpatient clinic, making it difficult to investigate their prognosis and causes of death.

## Conclusions

The prognosis of distal gastrectomy was better than that of non-surgery in patients aged ≥ 85 years. However, total gastrectomy should be avoided due to the high rate of postoperative pneumonia death.

## Data Availability

No datasets were generated or analysed during the current study.

## Abbreviations

ESD: endoscopic submucosal dissection
QOL: quality of life
ECOG PS: Eastern Cooperative Oncology Group performance status
POSSUM: Physiological and Operative Severity Score for the enUmeration of Mortality and morbidity
PNI: prognostic nutritional index
OS: Overall survival
